# Supplementary material for: Glycopeptide epitope facilitates HIV-1 envelope specific humoral immune responses by eliciting T cell help
Source: Nat Commun. 2020 May 21;11:2550. doi: 10.1038/s41467-020-16319-0 (PMC7242320; doi:10.1038/s41467-020-16319-0)
Supplement: Supplementary file 1 — Supplementary Information [file 41467_2020_16319_MOESM1_ESM.pdf]

Supplementary Information for

Glycopeptide epitope facilitates HIV-1 envelope specific humoral immune responses by eliciting T cell help

Sun et al.

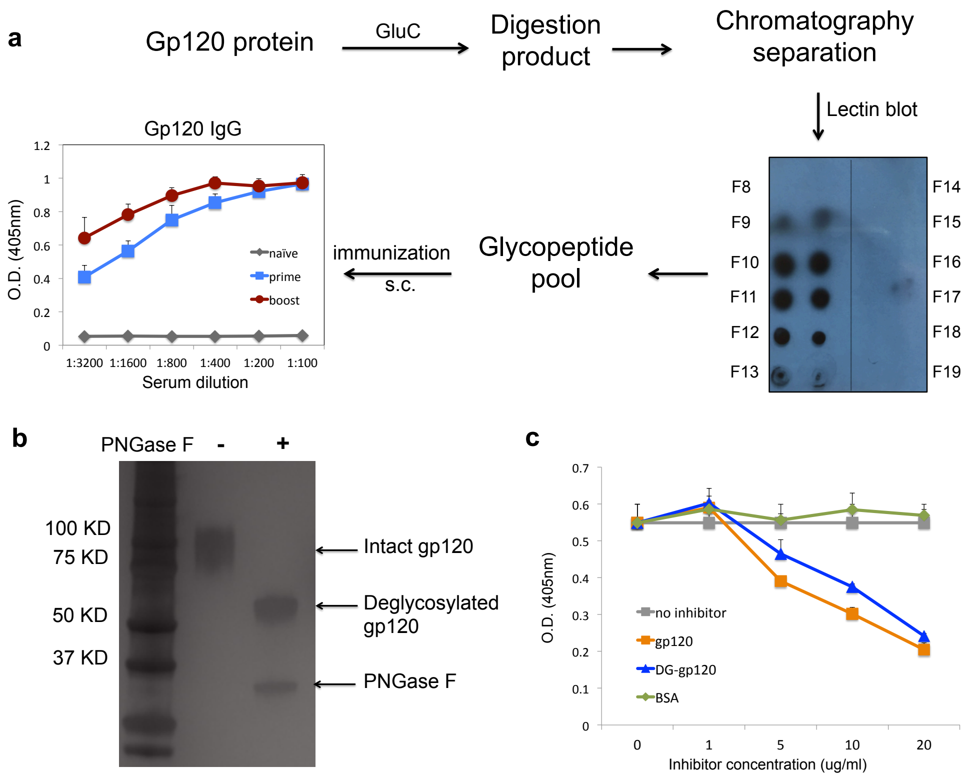

Supplementary Fig. 1. Generation of a gp120 glycopeptide pool and immunization scheme. (**a**) A pool of individual glycopeptides was produced from full-length gp120 with the use of protease Glu-C. Digested products were separated by chromatography. Glycopeptide-containing fractions were identified by lectin blot and were pooled together to immunize mice. Production of gp120-specific IgG was detected by ELISA. (**b**) gp120 was treated with PNGase F to remove N-glycans or was not treated. The molecular-weight shift of the proteins before and after glycosidase treatment are shown by Coomassie gel staining. (**c**) Recognition of coated DG-gp120 by antiserum from mice immunized with gp120 glycopeptides in the presence of the indicated inhibitors was examined by inhibition ELISA using a serum dilution 1:1600. Serum titers are reported as OD at 405 nm. Representative results are shown from one of three independent experiments performed. (mean±s.d.). **a** In the ELISA assay, *n* = 3 for naïve; *n* = 2 independent experiments for prime and boost. **c** *n* = 3 independent

experiments. *P*-values were determined using Student's 2-sided t-tests. Source data are provided as a Source Data file.

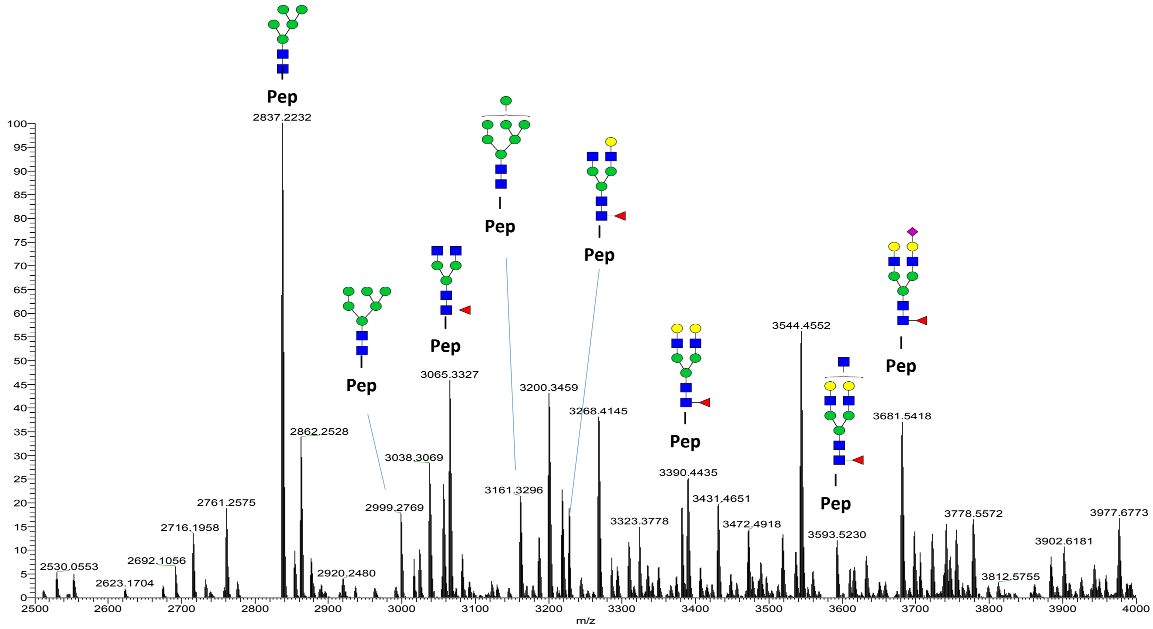

Supplementary Fig. 2. Glycopeptide profile of the GpepIP (LDVVPIDNNN$_{187}$TSYR) region in gp120 expressed in 293-F cells, as analyzed by MS. The original full MS spectrum (averaged spectrum) was obtained from 30 min to 40min of the LC-MS data, with the target glycopeptides eluted during the LC-MS run. The spectrum presented here shows data deconvoluted with Xtract software for quantification purposes.

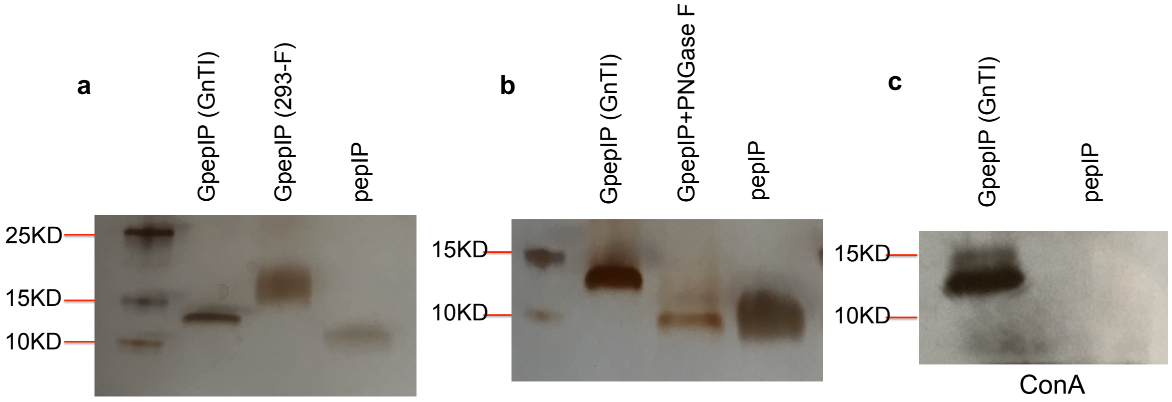

Supplementary Fig. 3. Characterization of three GpepIP variants. (**a**) GpepIP was recombinantly expressed in either FreeStyle™ 293-F cells or N-acetylglucosaminyltransferase I mutant (GnTI$^{-/-}$) cells. A peptide variant without glycan modification (pepIP) was chemically synthesized. The molecular weights of the three glycopeptides were determined by SDS-PAGE and silver staining. (**b**) GpepIP expressed in GnTI$^{-/-}$ cells was treated with PNGase F to remove N-glycans. (**c**) Glycosylation of GpepIP expressed in GnTI$^{-/-}$ cells and pepIP was detected by western blotting with the lectin ConA. Representative results are shown from one of three independent experiments performed. Source data are provided as a Source Data file.

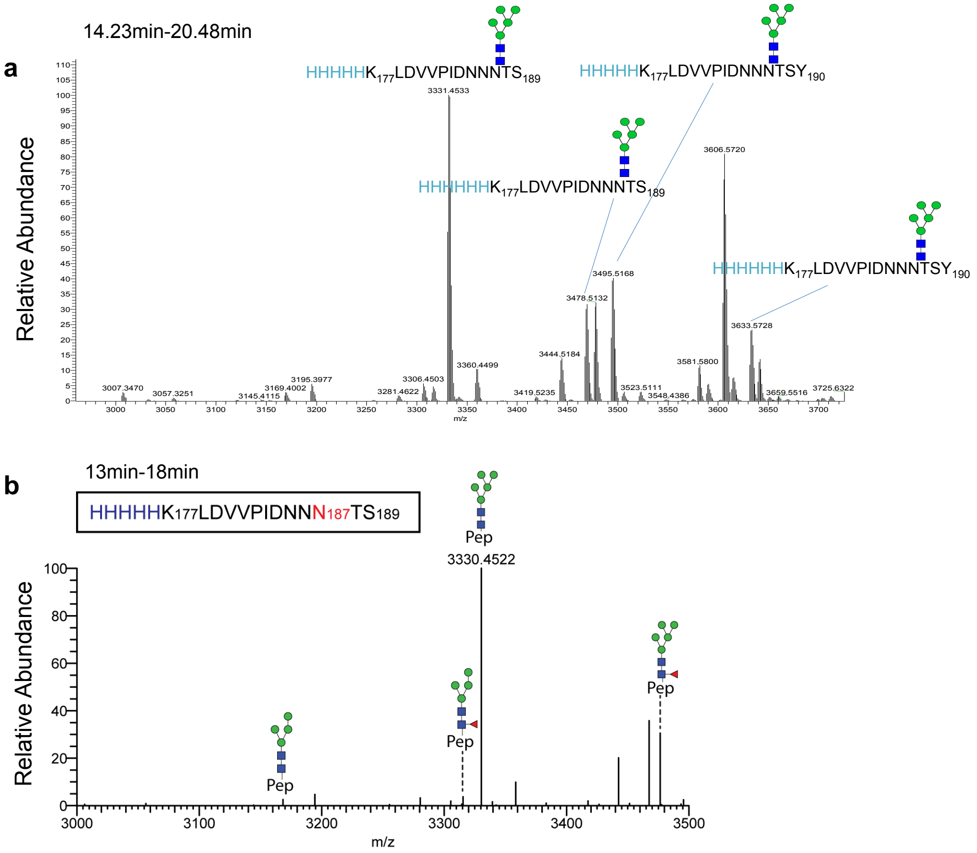

Supplementary Fig. 4. Glycopeptide profile of GpepIP expressed in GNTI$^{-/-}$ cells analyzed by MS. (**a**) The deconvoluted MS spectrum was obtained from the LC-MS time region 14.23–20.48 min. The peptide regions KLDVVPIDNNNTSY and KLDVVPIDNNNTS with His tagging (5 or 6 histidines) were identified. (**b**) Glycopeptides with the sequence HHHHHKLDVVPIDNNNTS were found between 13 min and 18 min in the LC-MS run. The deconvoluted spectrum from this time region is displayed. The major glycan observed is Hex5HexNAc2; Hex4HexNAc2, Hex5HexNAc2Fuc2, and Hex4HexNAc2Fuc2 were minor components. No complex-type structure or sialylated structure was detected.

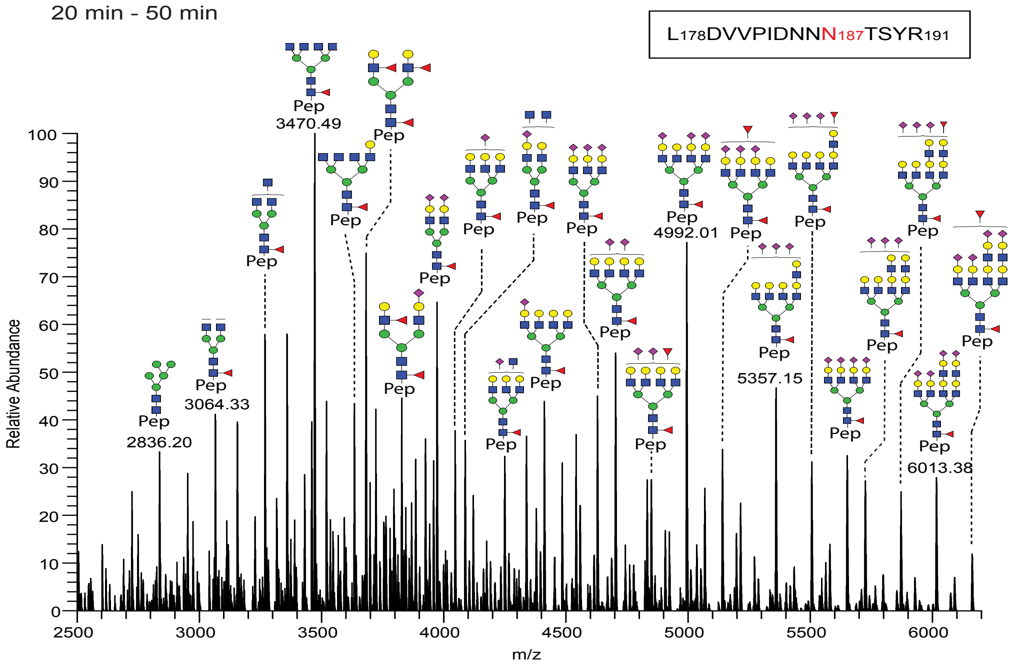

Supplementary Fig. 5. Full MS profile of N-glycans released from GpepIP expressed in 293-F cells. The glycopeptides from 293-F cells were eluted between 20 and 50 min in the LC-MS run, and the deconvoluted MS spectrum was obtained from this time region. Glycan composition of this glycopeptide showed that the recombinant glycopeptide carries more complex-type glycans with heavy fucosylation and sialylation, while high-mannose types were detected as minor components.

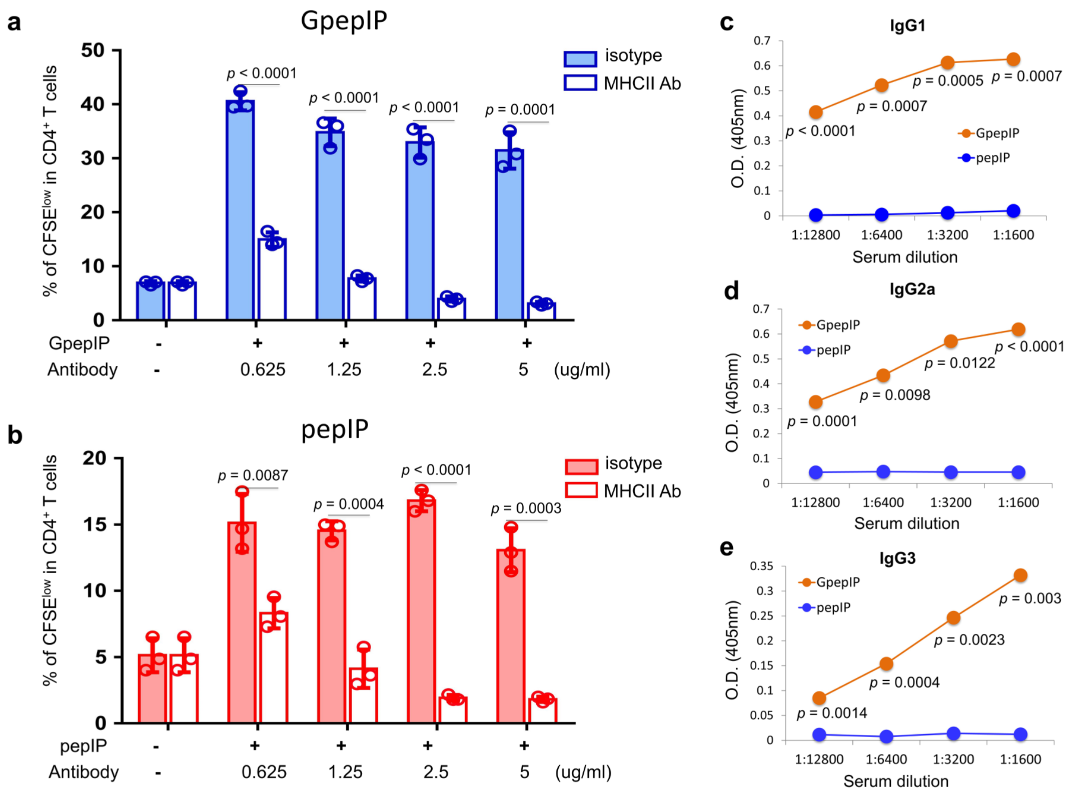

Supplementary Fig. 6. GpepIP- and pepIP-induced CD4 T cell responses are MHC-II-restricted. Splenocytes obtained from GpepIP (**a**) or pepIP (**b**) immunized mice were stimulated *in vitro* with GpepIP (**a**) or pepIP (**b**) respectively for 5 days in the presence of anti-MHCII antibody or isotype control at indicated concentration. T cell proliferation was examined by CFSE division by flow cytometry. (**c-e**) Serum from mice immunized with GpepIP expressed in GnT1$^{-/-}$ cells were collected 7 days after booster immunization. As a control, serum from naïve mice was used as background. Titers of IgG1 (**c**), IgG2a (**d**), and IgG3 (**e**) for recognition of GpepIP and pepIP were measured by ELISA. Representative results are shown from one of three independent experiments performed. (mean±s.d.). **a**, **b** n = 3 independent experiments. **c**, **d** and **e** n = 2 independent experiments. P-values were determined using Student's 2-sided t-tests. Source data are provided as a Source Data file.

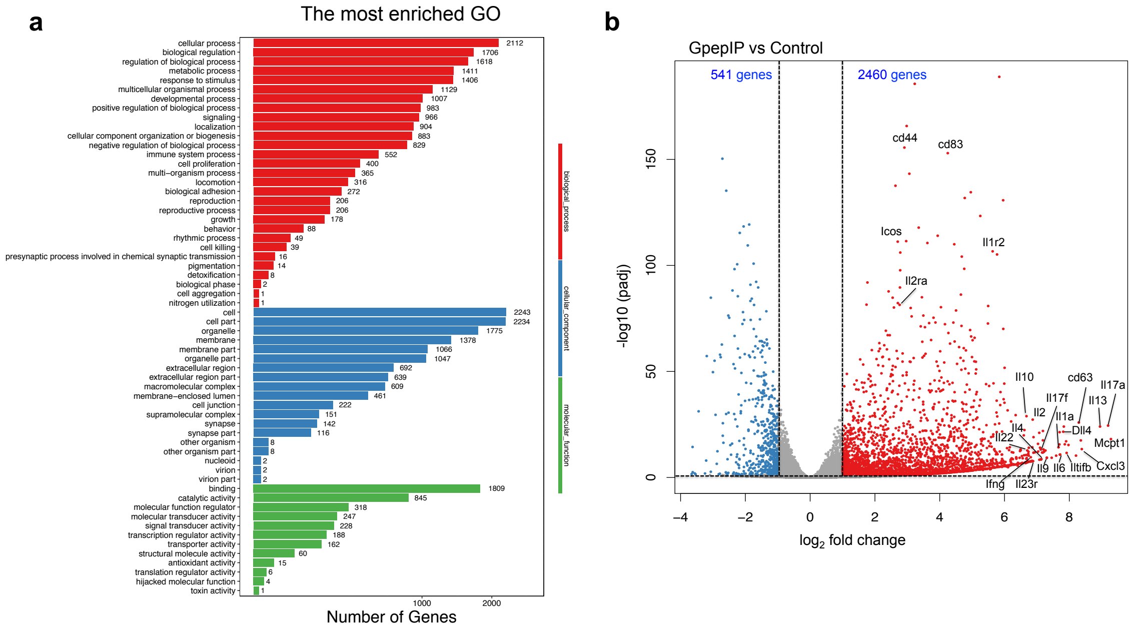

Supplementary Fig. 7. GO analysis and gene expression signature of GpepIP specific CD4$^+$ T cells. (**a**) The most enriched GO of DEGs in GpepIP specific CD4$^+$ T cells compared to control cells was represented. The number of genes of GO in biological process, cellular component and molecular function were shown. (**b**) Volcano plot showing the gene signature of GpepIP specific CD4$^+$ T cells compared control. DEGs (greater than twofold; P value < 0.05) were shown as colored dots. Genes associated with Th1 and Th2 signaling, Th17 signaling and activated T cell co-stimulatory molecules were labeled.

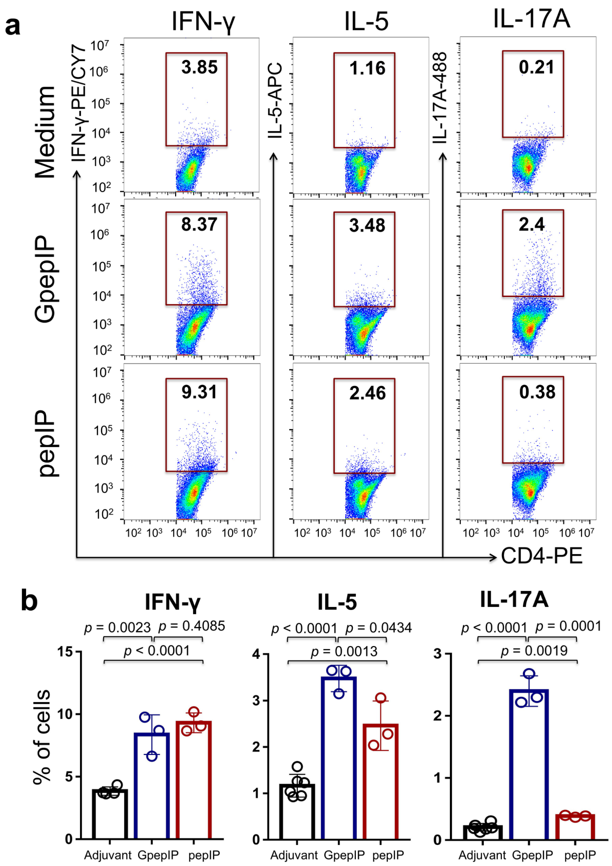

Supplementary Fig. 8. Th-cell related cytokine analysis of GpepIP- or pepIP-stimulated CD4$^+$ T cells. Splenic and lymph node cells isolated from GpepIP or pepIP immunized mice were stimulated with GpepIP or pepIP for 5 days. Cytokines IFN-γ, IL-5 and IL-17A on CD4$^+$ T cells were assessed by intracellular cytokine staining and flow cytometry. Representative results are shown from one of three independent experiments performed. (mean±s.d.). n = 6 for medium except for IFN-γ with n = 4; n = 3 independent experiments for GpepIP and pepIP. P-values were determined using Student's 2-sided t-tests. Source data are provided as a Source Data file.

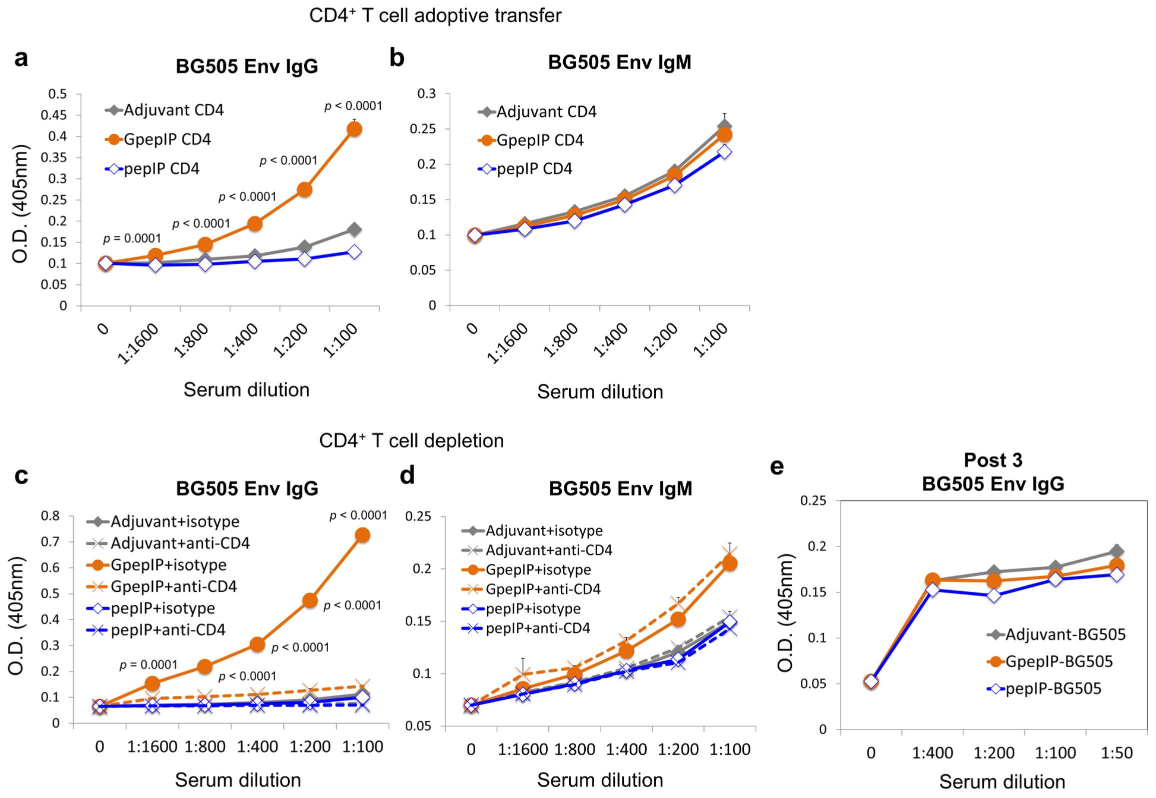

Supplementary Fig. 9. GpepIP epitope facilitates HIV Env trimer specific humoral immune responses dependent on CD4⁺ T cells. (**a, b**) BALB/c mice were immunized twice by subcutaneous injection of GpepIP or pepIP emulsified in Freund's adjuvant or of adjuvant alone. 5 days after boost immunization, CD4⁺ T cells were isolated from each group and adoptively transferred into naïve mice. The recipient mice were then immunized with the clade A BG505 gp140 NFL trimer 1 day after adoptive transfer. Sera were collected 7 days after trimer boost immunization. BG505-specific IgG (**a**) and IgM (**b**) production were examined by ELISA. (**c, d**) BALB/c mice were primed twice with GpepIP or pepIP emulsified in Freund's adjuvant or of adjuvant alone. Three weeks later, all groups were immunized with the clade A BG505 gp140 NFL trimer emulsified in incomplete Freund's adjuvant. For CD4⁺ T cell depletion, mice were i.p. injected with 250 µg either anti-CD4 or isotype control mAbs 3 days and 1 day before, and 2 days after trimer immunization. Sera were collected 7 days

after trimer boost immunization. BG505-specific IgG (**c**) and IgM (**d**) production were examined by ELISA. (**e**) BG505-specific IgG production was examined in antisera from adjuvant, GpepIP and pepIP primed and post the third trimer boost by ELISA. Representative results are shown from one of three independent experiments performed. (mean±s.d.). **a**, **b** *n* = 4 independent experiments except for 0 dilution with *n* = 6. **c**, **d** *n* = 3 independent experiments except for 0 dilution with *n* = 6. **e** *n* = 3 independent experiments except for 0 dilution with *n* = 4. *P*-values were determined using Student's 2-sided t-tests. Source data are provided as a Source Data file.

Supplementary Table 1. MHCII-bound gp120 glycopeptides/peptides

Protease used	Position	Sequence	Site of glycosylation	Observed mass (mass tolerance)	Charge state
No Enzyme	177-190	KLDVVPIDNNN@TSY	N187	797.89764 Da (+2.18 ppm)	2
	191-205	RLISC*DTSVITQ		696.86334 Da (+3.02 ppm)	2
Trypsin	48-60	EATTTLFC*ASDAK		707.83215 Da (+3.60 ppm)	2
	155-165	N@C*SFN@ITTSIR	N155, N159	659.80688 Da (-1.13 ppm)	2
	178-191	LDVVPIDNNN@TSYR	N187	811.90094 Da (+2.41 ppm)	2
	192-206	LISC*DTSVITQAC*PK		846.92218 Da (+4.43 ppm)	2
	289-303	ESVEIN@C*TRPNN@NTR	N294, N299	603.94348 D (-0.52 ppm),	3
	282-288	TIIVQLK		407.77341 Da (-0.2 ppm)	2
Chymotrypsin	178-190	LDVVPIDNNN@TSY	N187	733.85175 Da (+4.53 ppm)	2
	178-191	LDVVPIDNNN@TSYR	N187	811.90228 Da (+4.06 ppm)	2
	289-303	ESVEIN@C*TRPNN@NTR	N294, N299	603.94513 Da (+2.21 ppm)	3

C*: Carbamidomethylated cysteine; N@: ^{18}O labeled aspartic acid (site of N-glycosylation)
