# Supplementary material for: Glycopeptide epitope facilitates HIV-1 envelope specific humoral immune responses by eliciting T cell help
Source: Nat Commun. 2020 May 21;11:2550. doi: 10.1038/s41467-020-16319-0 (PMC7242320; doi:10.1038/s41467-020-16319-0)
Supplement: Supplementary file 3 — Reporting summary [file 41467_2020_16319_MOESM3_ESM.pdf]

nature research

Corresponding author(s): Fikri Y Avci

Last updated by author(s): Dec 28, 2019

Reporting Summary

Nature Research wishes to improve the reproducibility of the work that we publish. This form provides structure for consistency and transparency in reporting. For further information on Nature Research policies, see Authors & Referees and the Editorial Policy Checklist .

Statistics

For all statistical analyses, confirm that the following items are present in the figure legend, table legend, main text, or Methods section.

n/a	Confirmed	
☐	☒	The exact sample size (*n*) for each experimental group/condition, given as a discrete number and unit of measurement
☐	☒	A statement on whether measurements were taken from distinct samples or whether the same sample was measured repeatedly
☐	☒	The statistical test(s) used AND whether they are one- or two-sided<br>*Only common tests should be described solely by name; describe more complex techniques in the Methods section.*
☐	☒	A description of all covariates tested
☐	☒	A description of any assumptions or corrections, such as tests of normality and adjustment for multiple comparisons
☐	☒	A full description of the statistical parameters including central tendency (e.g. means) or other basic estimates (e.g. regression coefficient) AND variation (e.g. standard deviation) or associated estimates of uncertainty (e.g. confidence intervals)
☐	☒	For null hypothesis testing, the test statistic (e.g. *F*, *t*, *r*) with confidence intervals, effect sizes, degrees of freedom and *P* value noted<br>*Give P values as exact values whenever suitable.*
☒	☐	For Bayesian analysis, information on the choice of priors and Markov chain Monte Carlo settings
☐	☒	For hierarchical and complex designs, identification of the appropriate level for tests and full reporting of outcomes
☒	☐	Estimates of effect sizes (e.g. Cohen's *d*, Pearson's *r*), indicating how they were calculated

Our web collection on statistics for biologists contains articles on many of the points above.

Software and code

Policy information about availability of computer code

Data collection	Flow cytometry and multiplex-based cytokine analysis: CytoFLEX (Beckman Coulter, Hialeah, FL); ELISA data: microplate reader (Synergy H1, Bio-Tek); MS analysis: Orbitrap Fusion Lumos Tribrid mass spectrometer; Western blot: Odyssey CLx Imaging System (LI-COR Biosciences); RNA sequencing: BGISEQ-500 platform (Beijing Genomics Institute (BGI)
Data analysis	FlowJo V10.1 software (TreeStar) ; Graph Pad Prism V8 (GraphPad Software); LEGENDplex Software V7 (Biolegend); Proteome Discoverer 1.4 software (Thermo Fisher Scientific) and the TurboSequest algorithm; Byonic software (Protein Metrics); RSEM V1.2.12 69; Cluster V3.0; R language

For manuscripts utilizing custom algorithms or software that are central to the research but not yet described in published literature, software must be made available to editors/reviewers. We strongly encourage code deposition in a community repository (e.g. GitHub). See the Nature Research guidelines for submitting code & software for further information.

Data

Policy information about availability of data

All manuscripts must include a data availability statement. This statement should provide the following information, where applicable:

- Accession codes, unique identifiers, or web links for publicly available datasets
- A list of figures that have associated raw data
- A description of any restrictions on data availability

The source data underlying both the main and supplementary figures and uncropped gel images are provided as a Source Data file. All the mass spectrometry raw data generated for this study and Data S1-4 have been deposited in Mendeley Data repository with the identifier https://data.mendeley.com/datasets/b8sk9zc7xj/draft?a=c1f35f4d-a528-4f29-8418-bb641965944a (DOI will be obtained once manuscript accepted). All other data generated during the current study are available from the corresponding authors upon reasonable requests.

Field-specific reporting

Please select the one below that is the best fit for your research. If you are not sure, read the appropriate sections before making your selection.

- [x] Life sciences
- [ ] Behavioural & social sciences
- [ ] Ecological, evolutionary & environmental sciences

For a reference copy of the document with all sections, see nature.com/documents/nr-reporting-summary-flat.pdf

Life sciences study design

All studies must disclose on these points even when the disclosure is negative.

Sample size	No statistical methods were used to predetermine sample size. An estimate of three to ten samples per group were used in each experiment. Sample sizes were determined based on prior experience with similar experiments, or based on pilot experiments.<br>Samples in all experiments testing T cell proliferation in our manuscript (glycopeptide pool in Figure 1A, B; gp120 in Figure 3A, B, GpepIP, pepIP in Figure 3C, D, Figure 4 and 5; GpepIP/pepIP and BG505 in Figure 6B) are 4-6 per group collected from 2-3 experiments.<br>In testing Th associated cytokines in the cell culture supernatant (Figure 1 and Figure 5), four samples from each group are used and experiments are repeated twice.<br>For transcriptomic analysis (Figure 4), four mice from each group are immunized and cells are pooled into two samples per group.<br>In GpepIP/pepIP specific CD4+ T cell helping BG505 antibody response (Figure 6, 7), four samples are used and experiments are repeated twice.<br>For antibody neutralizing activity assay, two samples each group and three groups are examined.<br>Although in some experiments lesser sample size (< 4) is employed (as described above), the statistical difference between groups is readily detectable.
Data exclusions	No data were excluded in this study.
Replication	Most experiments performed in this study have repeated twice or three times as mentioned in "Sample size". All replications are successful.
Randomization	In animal studies, naive mice are randomly allocated for control and experimental groups. Immunized mice from control group and experiment group, or different immunogen groups are randomly kept in microisolator cages.
Blinding	Investigators carrying out neutralization assessment were blinded. For the rest of the experiments no blinding was deemed necessary as no subjective assessments were included.

Behavioural & social sciences study design

All studies must disclose on these points even when the disclosure is negative.

Study description	*Briefly describe the study type including whether data are quantitative, qualitative, or mixed-methods (e.g. qualitative cross-sectional, quantitative experimental, mixed-methods case study).*
Research sample	*State the research sample (e.g. Harvard university undergraduates, villagers in rural India) and provide relevant demographic information (e.g. age, sex) and indicate whether the sample is representative. Provide a rationale for the study sample chosen. For studies involving existing datasets, please describe the dataset and source.*
Sampling strategy	*Describe the sampling procedure (e.g. random, snowball, stratified, convenience). Describe the statistical methods that were used to predetermine sample size OR if no sample-size calculation was performed, describe how sample sizes were chosen and provide a rationale for why these sample sizes are sufficient. For qualitative data, please indicate whether data saturation was considered, and what criteria were used to decide that no further sampling was needed.*
Data collection	*Provide details about the data collection procedure, including the instruments or devices used to record the data (e.g. pen and paper, computer, eye tracker, video or audio equipment) whether anyone was present besides the participant(s) and the researcher, and whether the researcher was blind to experimental condition and/or the study hypothesis during data collection.*
Timing	*Indicate the start and stop dates of data collection. If there is a gap between collection periods, state the dates for each sample cohort.*
Data exclusions	*If no data were excluded from the analyses, state so OR if data were excluded, provide the exact number of exclusions and the rationale behind them, indicating whether exclusion criteria were pre-established.*
Non-participation	*State how many participants dropped out/declined participation and the reason(s) given OR provide response rate OR state that no participants dropped out/declined participation.*
Randomization	*If participants were not allocated into experimental groups, state so OR describe how participants were allocated to groups, and if allocation was not random, describe how covariates were controlled.*

Ecological, evolutionary & environmental sciences study design

All studies must disclose on these points even when the disclosure is negative.

Study description	*Briefly describe the study. For quantitative data include treatment factors and interactions, design structure (e.g. factorial, nested, hierarchical), nature and number of experimental units and replicates.*
Research sample	*Describe the research sample (e.g. a group of tagged Passer domesticus, all Stenocereus thurberi within Organ Pipe Cactus National Monument), and provide a rationale for the sample choice. When relevant, describe the organism taxa, source, sex, age range and any manipulations. State what population the sample is meant to represent when applicable. For studies involving existing datasets, describe the data and its source.*
Sampling strategy	*Note the sampling procedure. Describe the statistical methods that were used to predetermine sample size OR if no sample-size calculation was performed, describe how sample sizes were chosen and provide a rationale for why these sample sizes are sufficient.*
Data collection	*Describe the data collection procedure, including who recorded the data and how.*
Timing and spatial scale	*Indicate the start and stop dates of data collection, noting the frequency and periodicity of sampling and providing a rationale for these choices. If there is a gap between collection periods, state the dates for each sample cohort. Specify the spatial scale from which the data are taken*
Data exclusions	*If no data were excluded from the analyses, state so OR if data were excluded, describe the exclusions and the rationale behind them, indicating whether exclusion criteria were pre-established.*
Reproducibility	*Describe the measures taken to verify the reproducibility of experimental findings. For each experiment, note whether any attempts to repeat the experiment failed OR state that all attempts to repeat the experiment were successful.*
Randomization	*Describe how samples/organisms/participants were allocated into groups. If allocation was not random, describe how covariates were controlled. If this is not relevant to your study, explain why.*
Blinding	*Describe the extent of blinding used during data acquisition and analysis. If blinding was not possible, describe why OR explain why blinding was not relevant to your study.*

Did the study involve field work? ☐ Yes ☐ No

Field work, collection and transport

Field conditions	*Describe the study conditions for field work, providing relevant parameters (e.g. temperature, rainfall).*
Location	*State the location of the sampling or experiment, providing relevant parameters (e.g. latitude and longitude, elevation, water depth).*
Access and import/export	*Describe the efforts you have made to access habitats and to collect and import/export your samples in a responsible manner and in compliance with local, national and international laws, noting any permits that were obtained (give the name of the issuing authority, the date of issue, and any identifying information).*
Disturbance	*Describe any disturbance caused by the study and how it was minimized.*

Reporting for specific materials, systems and methods

We require information from authors about some types of materials, experimental systems and methods used in many studies. Here, indicate whether each material, system or method listed is relevant to your study. If you are not sure if a list item applies to your research, read the appropriate section before selecting a response.

Materials & experimental systems

n/a	Involved in the study	
☐	☒	Antibodies
☐	☒	Eukaryotic cell lines
☒	☐	Palaeontology
☐	☒	Animals and other organisms
☒	☐	Human research participants
☒	☐	Clinical data

Methods

n/a	Involved in the study	
☒	☐	ChIP-seq
☐	☒	Flow cytometry
☒	☐	MRI-based neuroimaging

Antibodies

Antibodies used	For MHCII blocking:<br>anti-mouse I-A/I-E antibody (Biolegend, clone M5/114.15.2, catalog number 107601) at the indicated concentration<br>Rat IgG2b isotype control (Biolegend, clone RTK4530, catalog number 400644) at the indicated concentration<br><br>For flow cytometry detection, the following fluorophore-conjugated antibodies were purchased from BioLegend:<br>anti-mouse CD4 (dilution 1:3000, clone GK1.5, catalog number 100407)<br>anti-mouse CD4 (dilution 1:3000, clone RM4-4, catalog number 116013)<br>anti-mouse/human CD45R/B220 (dilution 1:2000, clone RA3-6B2, catalog number 103224)<br>anti-mouse/human GL7 (dilution 1:400, clone GL7, catalog number 144612)<br>anti-mouse CD95 (Fas) (dilution 1:400, clone SA367H8, catalog number 152603)<br>anti-mouse CD69 (dilution 1:1600, clone H1.2F3, catalog number 104507)<br>anti-mouse CD80 (dilution 1:1000, clone 16-10A1, catalog number 104707)<br>anti-mouse I-A/I-E (dilution 1:2000, clone M5/114.15.2, catalog number 107615)<br>anti-mouse CD11c antibody (dilution 1:500, clone N418, catalog number 117318)<br><br>For CD4 depletion:<br>anti-mouse CD4 (Biolegend, clone GK1.5, catalog number 100457)<br>Rat IgG2b isotype control (Biolegend, clone RTK4530, catalog number 400671)<br><br>For intracellular staining, the following antibodies were purchased from Biolegend:<br>anti-mouse IFN-γ (dilution 1:200, clone XMG1.2, catalog number 505825)<br>anti-mouse/human IL-5 (dilution 1:200, clone TRFK5, catalog number 504305)<br>anti-mouse IL-17A (dilution 1:400, clone TC11-18H10.1, catalog number 506909)<br><br>For ELISA:<br>Purified anti-mouse IFN-γ Antibody (Biolegend, dilution 1:1000, clone XMG1.2, catalog number 505812)<br>Biotin anti-mouse IFN-γ Antibody (Biolegend, dilution 1:1000, clone R4-6A2, catalog number 505704)<br>Purified anti-mouse IL-4 Antibody (Biolegend, dilution 1:1000, clone 11B11, catalog number 504115)<br>Biotin-labeled anti-mouse IL-4 antibody (Biolegend, dilution 1:1000, clone BVD6-24G2, catalog number 504202)<br>HRP-conjugated Avidin (Biolegend dilution 1:1000, catalog number 405103)<br>anti-gp120 mAb D7324 (Aalto Bioreagents, Dublin, Irelanddilution 1:200, catalog number D7324)<br>alkaline phosphatase (AP)–conjugated anti-mouse IgG (Southern Biotech, dilution 1:2000, catalog number 1030-04)<br>HRP-conjugated anti-mouse IgG1 (Abcam, dilution 1:10000, catalog number ab97240)<br>HRP-conjugated anti-mouse IgG2a (Abcam, dilution 1:10000, catalog number ab97245)<br>HRP-conjugated anti-mouse IgG2b (Abcam, dilution 1:10000, catalog number ab97250)<br>HRP-conjugated anti-mouse IgG3 (Abcam, dilution 1:10000, catalog number ab97260)
Validation	All antibodies in this study (assay and species) were commercially purchased and have been validated by the vendors. Validation data are available from the respective vendor's respective websites.

Eukaryotic cell lines

Policy information about cell lines

Cell line source(s)	Under Materials and Methods, section: Antigen production.<br>FreeStyle 293-F suspension cells were obtained from Life Technologies (Catalog number: R79007), 293T cells are from ATCC (Catalog number CRL-3216) and HEK293S GnTI- /- cells are from ATCC (Catalog number: CRL-3022).
Authentication	Cell lines are authenticated by the following methods:<br>1. Morphology check. We authenticate the cell lines by observing their cellular morphology under the microscope referring to ATCC standards.<br>2. Growth curve analysis. We monitor and record the cell proliferation rate and make sure the cells are growing in a stable and consistent rate. And by doing this, we optimize the best time to subculture and the optimum dilution at various cell densities.<br>3. Indirectly by evaluating antigen glycosylation. By profiling the glycan structure of antigens produced in 293-F and GnTI-/- cells by mass spectrometry, we confrimed that antigens produced in 293-F cells are almost exclusively complex-type glycans while antigens from GnTI-/- cells are exclusively oligomannose N-glycans, which are consistent with literature reports.
Mycoplasma contamination	The cell lines have been tested negative for mycoplasma contamination by PCR-based "Universal Mycoplasma Detection Kit (ATCC 30-1012K)".
Commonly misidentified lines<br>(See ICLAC register)	No cell lines used in this paper are listed in the database of misidentified cell lines in ICLAC.

Palaeontology

Specimen provenance | *Provide provenance information for specimens and describe permits that were obtained for the work (including the name of the issuing authority, the date of issue, and any identifying information).*

Specimen deposition | *Indicate where the specimens have been deposited to permit free access by other researchers.*

Dating methods | *If new dates are provided, describe how they were obtained (e.g. collection, storage, sample pretreatment and measurement), where they were obtained (i.e. lab name), the calibration program and the protocol for quality assurance OR state that no new dates are provided.*

☐ Tick this box to confirm that the raw and calibrated dates are available in the paper or in Supplementary Information.

Animals and other organisms

Policy information about studies involving animals; ARRIVE guidelines recommended for reporting animal research

Laboratory animals	Eight-week-old female BALB/c mice were obtained from The Jackson Laboratory (Bar Harbor, ME) and housed in the Center for Molecular Medicine Rodent Vivarium at the University of Georgia with housing condition of 20°C temperature and 60% humidity.
Wild animals	No wild animals were used in the study.
Field-collected samples	No field-collected samples were used in the study.
Ethics oversight	All mouse experiments were conducted in compliance with the University of Georgia Institutional Animal Care and Use Committee under an approved animal use protocol."

Note that full information on the approval of the study protocol must also be provided in the manuscript.

Human research participants

Policy information about studies involving human research participants

Population characteristics | *Describe the covariate-relevant population characteristics of the human research participants (e.g. age, gender, genotypic information, past and current diagnosis and treatment categories). If you filled out the behavioural & social sciences study design questions and have nothing to add here, write "See above."*

Recruitment | *Describe how participants were recruited. Outline any potential self-selection bias or other biases that may be present and how these are likely to impact results.*

Ethics oversight | *Identify the organization(s) that approved the study protocol.*

Note that full information on the approval of the study protocol must also be provided in the manuscript.

Clinical data

Policy information about clinical studies

All manuscripts should comply with the ICMJEguidelines for publication of clinical research and a completedCONSORT checklist must be included with all submissions.

Clinical trial registration | *Provide the trial registration number from ClinicalTrials.gov or an equivalent agency.*

Study protocol | *Note where the full trial protocol can be accessed OR if not available, explain why.*

Data collection | *Describe the settings and locales of data collection, noting the time periods of recruitment and data collection.*

Outcomes | *Describe how you pre-defined primary and secondary outcome measures and how you assessed these measures.*

ChIP-seq

Data deposition

☐ Confirm that both raw and final processed data have been deposited in a public database such as GEO.

☐ Confirm that you have deposited or provided access to graph files (e.g. BED files) for the called peaks.

Data access links
May remain private before publication. | *For "Initial submission" or "Revised version" documents, provide reviewer access links. For your "Final submission" document, provide a link to the deposited data.*

Files in database submission | *Provide a list of all files available in the database submission.*

Genome browser session
(e.g. UCSC)

Provide a link to an anonymized genome browser session for "Initial submission" and "Revised version" documents only, to enable peer review. Write "no longer applicable" for "Final submission" documents.

Methodology

Replicates	*Describe the experimental replicates, specifying number, type and replicate agreement.*
Sequencing depth	*Describe the sequencing depth for each experiment, providing the total number of reads, uniquely mapped reads, length of reads and whether they were paired- or single-end.*
Antibodies	*Describe the antibodies used for the ChIP-seq experiments; as applicable, provide supplier name, catalog number, clone name, and lot number.*
Peak calling parameters	*Specify the command line program and parameters used for read mapping and peak calling, including the ChIP, control and index files used.*
Data quality	*Describe the methods used to ensure data quality in full detail, including how many peaks are at FDR 5% and above 5-fold enrichment.*
Software	*Describe the software used to collect and analyze the ChIP-seq data. For custom code that has been deposited into a community repository, provide accession details.*

Flow Cytometry

Plots

Confirm that:

- [x] The axis labels state the marker and fluorochrome used (e.g. CD4-FITC).
- [x] The axis scales are clearly visible. Include numbers along axes only for bottom left plot of group (a 'group' is an analysis of identical markers).
- [x] All plots are contour plots with outliers or pseudocolor plots.
- [x] A numerical value for number of cells or percentage (with statistics) is provided.

Methodology

Sample preparation	Single cell suspensions from LNs and spleens were generated by gently grinding the tissue between sterile glass slides. Bone marrow was flushed out from the tibiae and femurs of 6-8-week-old female BALB/c mice.
Instrument	CytoFLEX (Beckman Coulter, Hialeah, FL)
Software	FlowJo (Tree Star, Inc., Ashland, OR)
Cell population abundance	1-2 million antigen-specific CD4+ T cells were sorted per group for RNA-seq experiments. Purity was assessed by staining for CD4 and CD69 marker using flow cytometry.
Gating strategy	The cells were first gated on the lymphocyte population on the FSC/SSC plot. Singlets were gated on FSC-A/FSC-W plot. Thereafter CD4 T cells or B cells were determined based on staining for CD4 or B220. Subsequent characteristics were analyzed within these population. BMDC were dertermined based on staining of CD11c.

- [x] Tick this box to confirm that a figure exemplifying the gating strategy is provided in the Supplementary Information.

Magnetic resonance imaging

Experimental design

Design type	*Indicate task or resting state; event-related or block design.*
Design specifications	*Specify the number of blocks, trials or experimental units per session and/or subject, and specify the length of each trial or block (if trials are blocked) and interval between trials.*
Behavioral performance measures	*State number and/or type of variables recorded (e.g. correct button press, response time) and what statistics were used to establish that the subjects were performing the task as expected (e.g. mean, range, and/or standard deviation across subjects).*

Acquisition

Imaging type(s) | *Specify: functional, structural, diffusion, perfusion.*

Field strength | *Specify in Tesla*

Sequence & imaging parameters | *Specify the pulse sequence type (gradient echo, spin echo, etc.), imaging type (EPI, spiral, etc.), field of view, matrix size, slice thickness, orientation and TE/TR/flip angle.*

Area of acquisition | *State whether a whole brain scan was used OR define the area of acquisition, describing how the region was determined.*

Diffusion MRI ☐ Used ☐ Not used

Preprocessing

Preprocessing software | *Provide detail on software version and revision number and on specific parameters (model/functions, brain extraction, segmentation, smoothing kernel size, etc.).*

Normalization | *If data were normalized/standardized, describe the approach(es): specify linear or non-linear and define image types used for transformation OR indicate that data were not normalized and explain rationale for lack of normalization.*

Normalization template | *Describe the template used for normalization/transformation, specifying subject space or group standardized space (e.g. original Talairach, MNI305, ICBM152) OR indicate that the data were not normalized.*

Noise and artifact removal | *Describe your procedure(s) for artifact and structured noise removal, specifying motion parameters, tissue signals and physiological signals (heart rate, respiration).*

Volume censoring | *Define your software and/or method and criteria for volume censoring, and state the extent of such censoring.*

Statistical modeling & inference

Model type and settings | *Specify type (mass univariate, multivariate, RSA, predictive, etc.) and describe essential details of the model at the first and second levels (e.g. fixed, random or mixed effects; drift or auto-correlation).*

Effect(s) tested | *Define precise effect in terms of the task or stimulus conditions instead of psychological concepts and indicate whether ANOVA or factorial designs were used.*

Specify type of analysis: ☐ Whole brain ☐ ROI-based ☐ Both

Statistic type for inference
(See Eklund et al. 2016) | *Specify voxel-wise or cluster-wise and report all relevant parameters for cluster-wise methods.*

Correction | *Describe the type of correction and how it is obtained for multiple comparisons (e.g. FWE, FDR, permutation or Monte Carlo).*

Models & analysis

n/a	Involved in the study
☐	☐ Functional and/or effective connectivity
☐	☐ Graph analysis
☐	☐ Multivariate modeling or predictive analysis

Functional and/or effective connectivity | *Report the measures of dependence used and the model details (e.g. Pearson correlation, partial correlation, mutual information).*

Graph analysis | *Report the dependent variable and connectivity measure, specifying weighted graph or binarized graph, subject- or group-level, and the global and/or node summaries used (e.g. clustering coefficient, efficiency, etc.).*

Multivariate modeling and predictive analysis | *Specify independent variables, features extraction and dimension reduction, model, training and evaluation metrics.*
